# Evaluating the Chemical Reactivity of Wildfire-Derived Dissolved Organic Molecules: Glutathione Binding through Kendrick Mass Defect Analysis

**DOI:** 10.1021/jasms.5c00077

**Published:** 2025-05-13

**Authors:** Hannah M. Hamontree, Patrick G. Hatcher

**Affiliations:** Department of Chemistry & Biochemistry, 6042 Old Dominion University, 4501 Elkhorn Ave, Norfolk, Virginia 23529, United States of America

**Keywords:** pyrogenic-derived dissolved organic matter, wildfire, Kendrick Mass Defect analysis

## Abstract

The emerging risks to organisms of pyrogenic-derived dissolved organic matter (PyDOM) from forest fires are of concern due to its toxic and mutagenic potential (e.g., pro-oxidative responses in fauna through the depletion of glutathione, a nitrogen- and sulfur-containing tripeptide found in cells). This study simulates this phenomenon in a laboratory setting by identifying bonding between reduced l-glutathione and organic molecules in leachates from environmentally weathered biomass samples (charred and uncharred) using Kendrick Mass Defect (KMD) analysis from formula lists obtained from negative-mode electrospray ionization-Fourier transform-ion cyclotron resonance-mass spectrometry ((−)­ESI-FT-ICR-MS). These formula lists reveal a 10-fold increase in nitrogen- and sulfur-containing molecular formulas in the charred biomass samples compared with the unreacted charred biomass when subjected to reaction with glutathione. KMD analysis attributed the bonding of glutathione to the biomass leachates accounting for approximately 25% of the new nitrogen- and sulfur-containing molecular formulas as either addition-type or condensation/elimination-type reactions. KMD sheds light on a different fraction of chemically reactive wildfire-produced organic compounds that may be of interest for subsequent toxicological studies.

## Graphical abstract

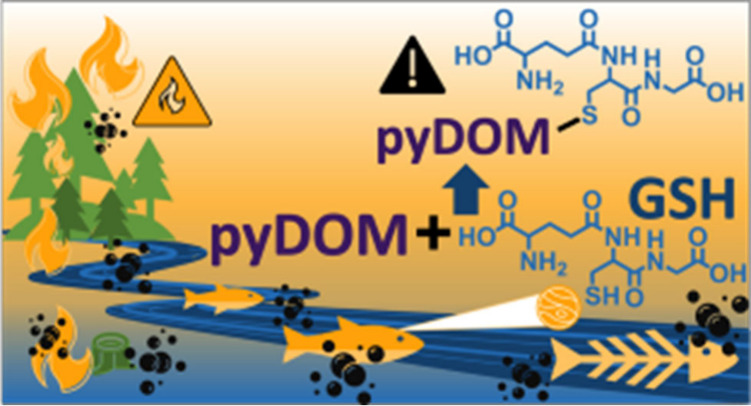

## Introduction

Pyrogenic carbon (PyC) is produced in the natural environment following wildfire events from the incomplete combustion of biomass (e.g., vegetation and soil organic matter). These molecules, characterized by their condensed aromatic content, can become solubilized and enter aquatic environmental systems when exposed to rain events, thus contributing to the pool of pyrogenic-derived dissolved organic matter (PyDOM) in riverine systems.^1^ PyDOM can be mobilized 5 to 15 years following major burn events^2^ and is estimated to contribute 18 ± 4 Tg PyC per year to riverine dissolved organic carbon (DOC) transport.^3^ Studying the uptake and exposure of dissolved PyC to higher organisms is important in evaluating overall ecosystem health due to the global presence, abundance, longevity, and propensity of wildfire occurrence—and PyC production—in the coming decades.

The production, transport, and ingestion of soluble polycyclic aromatic hydrocarbons (PAHs) in PyDOM are of environmental and biological concern due to their toxic, mutagenic, carcinogenic potential, and ability to bioaccumulate.^4^ Current toxicological studies focus on the influence of PAHs and metals to explain the observed toxicity and inhibitory effects in aquatic fauna exposed to wildfire ash and runoff.^5–8^ Such studies investigate oxidative stress using the biomarker glutathione (GSH), a thiol-containing tripeptide whose depletion in cells can be an indicator of toxicity and cell viability.^9^ These PAHs are subjected to oxidative attack by reactive oxygen species (ROS) in organisms and form oxygenated PAHs which can react with GSH via numerous covalent bonding reactions (e.g., the Michael reaction or formation of Schiff bases with ketones or quinones).^10^ Through these studies, no correlation exists between PAH or metal concentrations with glutathione enzymatic effects—begging the question “What additional PyDOM components must be considered to elucidate a correlation between PyDOM and organismal toxicity?”

We propose that the highly complex matrix of PyDOM contains additional compounds other than the well-known PAHs which can traverse the cellular membrane, deplete GSH through covalent interactions, and subsequently trigger pro-oxidative responses in aquatic fauna. Studying the effects of more complex PyDOM molecules is challenging due to the limited techniques available. However, studies do exist which examine emerging risks to organisms from biochar—a similarly complex, yet physiochemically different substance to wildfire PyC. The few studies which investigate the risks of laboratory-generated biochar aqueous eluates (anthropogenic slow-pyrolysis PyC or hydrothermal carbonization hydrochar) indicate organismal toxicity and provide suggestive evidence that biochar aqueous eluates can traverse the cellular membrane and induce toxicity in organisms.^11–14^ Unfortunately, these biochar studies have their limitations as slow-pyrolysis biochar is not a well suited proxy for naturally produced wildfire PyC due to physiochemical differences.^15,16^

This study acts as a preliminary screening of the toxic potential of PyDOM to aqueous fauna in a laboratory setting by promoting covalent bonding of GSH to PyDOM molecules to simulate the pro-oxidative stress which occurs through the depletion of GSH. We propose that GSH covalent bonding can occur with a wide chemical variety of PyDOM molecules and demonstrate chemical reactivity analytically via ultrahigh resolution mass spectrometry (negative-mode electrospray ionization (−)­ESI-FT-ICR-MS—an analytical technique which was previously employed to demonstrate that quinones produced from oxidized plant-borne phenolic molecules (e.g., tocopherols) can bond with GSH to induce cell death and toxicity).^17–19^ The high mass accuracy of the FT-ICR-MS identifies the bonding of glutathione to PyDOM molecules leached from their respective PyC biomasses using Kendrick Mass Defect analysis.

## Materials and Methods

### Environmental Sample Set

Three environmental samples were collected from a historical burn site (Blackwater Ecological Preserve, Zuni, VA, USA). These samples include uncharred (no wildfire exposure) pine wood, charred (wildfire-exposed) pine wood, and charred pine bark—the latter two were produced from a controlled burn and aged naturally as wildfire-generated PyC biomass for seven months, after which they were collected.

### Sample Preparation: Dissolved Organic Matter (DOM) Leachates

The uncharred pine wood, charred pine wood, and charred pine bark were dried, homogenized, ground with a mortar and pestle to a fine powder, and leached by adding Milli-Q Nanopure water (18.1 MΩ) to the sample in a 40:1 ratio (mL:g) and agitated on a platform shaker at 60 rpm for 50 h in darkness. The resulting dissolved organic matter (DOM) leachates were isolated and filtered using PTFE Syringe Filters (hydrophilic 0.22 μm particle retention, Titan3).

### Sample Preparation: GSH-DOM Reaction

The synthesis of the GSH-DOM adducts was modified from the method of Briggs et al.^20^ in which the monosodium salt of GSH (l-glutathione reduced, Sigma-Aldrich, G4251) was dissolved in hot ethanol^19^ and added to acidified DOM leachates (pH 2) in a 1:3 ratio (mg C DOC: mg C GSH) under a nitrogen stream and left in darkness for 117 h at room temperature.

### Instrumental Analysis of DOM Leachates and GSH-Reacted DOM Leachates

Elemental analysis (carbon %, nitrogen %, and hydrogen %) was performed on the environmental samples, and the dissolved organic carbon (DOC) content was reported for the respective DOM leachates (SI Section 1). Bulk structural characterization of the solid biomass was performed using solid-state ^13^C nuclear magnetic resonance spectroscopy (SI Section 2.1). Structural characterization of the solid samples and their respective DOM leachates is detailed in the Supporting Information.

### Liquid-State ^1^H Nuclear Magnetic Resonance (NMR)

One-dimensional (1D) ^1^H spectra were acquired via the PEW5shapepr pulse program with a relaxation delay of 4 s; a free induction decay of 10 k was zero-filled to a 16 k-sized data set and apodized with a 3-Hz Lorentzian window function. Sample DOM leachates were diluted with deuterated water to produce a 90:10 H_2_O:D_2_O solution. As an internal reference, sodium 2,2,3,3-tetraheutero-3-trimethylsilylpropanoate (TMSP) was added. Liquid-state analyses were performed at room temperature on a 400 MHz (9.4 T) Bruker BioSpin AVANCE III spectrometer at the Old Dominion University College of Sciences Major Instrumentation Cluster (COSMIC) facility using a double-resonance broadband z-gradient inverse (BBI) probe and processed with Bruker TopSpin software. Methanol (δ = 3.34 ppm) was used to internally calibrate the spectra due to its distinguishable singlet. The spectra were then phased and baseline-corrected. In addition, the water suppression technique used for these water extracts blanks out the signals in the region of 4.7–5.0 ppm.

### Electrospray Ionization-Fourier Transform-Ion Cyclotron Resonance-Mass Spectrometry (ESI-FT-ICR-MS)

FT-ICR-MS was performed for molecular-level characterization of the DOM leachates and GSH-reacted DOM leachates. The pH 2 unreacted and GSH-reacted DOM leachates were solid phase extracted using activated (3 cartridge volumes methanol and 6 cartridge volumes of pH 2 Milli-Q water) PPL cartridges.^21^ Once loaded, samples were rinsed with 3 cartridge volumes of pH 2 Milli-Q water and dried under a stream of nitrogen gas. Immediately prior to FT-ICR-MS analysis, samples were eluted with 1.0–2.0 mL of methanol to obtain a DOM eluate of approximately 20 ppm of C.

The eluates were analyzed using an Apollo II electrospray ionization source coupled to a Bruker Daltonics 10 T Apex Qe FT-ICR-MS (Old Dominion University College of Sciences Major Instrumentation Cluster COSMIC facility) in negative ion mode with a direct injection rate of 120 μL h^–1^ and an argon dry gas flow rate of 5.0 L min^–1^. Negative ion mode was chosen as it favors the detection of acidic functional groups,^22^ which are typically prevalent in PyDOM samples as carboxylic acids, allowing for the better observation of the formation of molecular bonds between DOM and GSH (via the thiol or amine), whereas positive ion mode favors the detection of aliphatic and carbohydrate-like molecules^23^ which typically do not contain functionalities as conducive with sulfur or nitrogen incorporation. ESI voltages were optimized for each sample with the spray shield voltage varying within ±400 V (3200–3600 V) and the capillary voltage varying within ±600 V (3600–4200) to achieve a consistent ion current ensuring similar target spectra magnitude of 1 × 10^7^ among all samples with the capillary temperature set to 200 °C. The acquisition had a source accumulation time (H_1_) of 0.001 s, ion accumulation time (H_2_) of 0.2 s, a time-of-flight of 0.0009 s, and an excitation amplitude of 8.75 V. Each run was acquired in broadband mode (200–800 Da) and co-added exactly 300 transients (i.e., number of scans) using a 4 Mega-Word time domain. Summed free induction decay signals were zero-filled once and Sine-Bell apodized prior to fast Fourier transformation and magnitude calculation using Bruker Daltonics Apex Control Software.

All samples were externally calibrated with a polyethylene glycol polymer standard for accurate *m*/*z* measurements (200–800 Da) and internally calibrated with a fatty acid and homologous compound series present in each spectrum^24^ using Bruker Daltonics Data Analysis Software. The TEnvR MATLAB script was used for data processing.^25^ Blank, salt, and isotopologue (^37^Cl, ^13^C) peaks were removed, and FT-ICR-MS spectral peaks were assigned a molecular formula within ±1 ppm error according to the following elemental composition parameters: ^12^C_5–∞_, ^1^H_5–100_, ^16^O_1–30_, ^14^N_0–5_, and ^32^S_0–2_. Formulas were refined according to previously established rules^26^ and through the inclusion within homologous series so that at least 90% of the mass spectral peaks were assigned.^27,28^ Formulas were plotted on a Van Krevelen diagram to compare formulas based on their hydrogen-to-carbon (H/C) content and their oxygen-to-carbon (O/C) content.^29^ The diagram is a useful tool to visualize compound classes (e.g., lipids, proteins, amino sugars, carbohydrates, lignin, oxidized lignin/tannins, and condensed aromatics). Additional formulas were removed on a case-by-case basis based on the structural formula possibilities (outliers in van Krevelen diagrams).

### Kendrick Mass Defect Analysis of DOM Leachates and GSH-Reacted Leachates

Kendrick Mass Defect (KMD) analysis generates a series of related compounds by identifying repeating structural moieties (e.g., CH_2_, COO, GSH, etc.) between specific formulas.^30^ The application of this process using GSH verifies that the only difference between two molecular formulas in the series is the addition of GSH (C_10_H_17_O_6_N_3_S_1_). The addition-type KMD analysis generates a series of GSH-bonded formulas using the exact mass of GSH (*m*/*z* 307.083805), Kendrick Mass Ratio (KMR) of 0.999727 (quotient of 307 divided by 307.083805), Kendrick Nominal Mass (KNM, molecular formulas normalized to Kendrick Mass Ratio of GSH), and Kendrick Mass Defect (KMD, difference between formulas’s KNM and nominal mass). A second KMD series was performed to verify covalent bonding through a condensation/elimination-type (e.g., Schiff base) reaction in which the difference between two molecular formulas was the addition of GSH (C_10_H_17_O_6_N_3_S_1_) and loss of water (H_2_O) using the Kendrick Mass Ratio of 0.999746 (quotient of 289 divided by 289.073244).

Once processed, a plot of KMD versus KNM demonstrates formulas with a similar KMD series (horizontal dislocation, slope of 0, and KNM difference of 307 amu). Molecular formula pairs that met this criterion were considered for the addition-type KMD series of GSH covalently bonding to the samples. The same procedures were also applied to the condensation/elimination-type (e.g.,Schiff base) KMD series. Additionally, the KMD series formulas were manually reviewed to ensure that each corresponding pair differed by the correct number of carbon, hydrogen, oxygen, nitrogen, and sulfur atoms and that the peaks were Lorentzian, high relative magnitude (≥106), and contained a signal-to-noise ≥3. An example of the relative magnitude and signal-to-noise of the KMD series pair before and after GSH binding (i.e., precursor and product, respectively) of the FT-ICR-MS spectra peaks is given in Figure S3 and Table S4. These data show that the peaks for the sample materials are significantly intense both before and after GSH incorporation.

## Results and Discussion

### FT-ICR-MS of Environmental Sample Set Leachates

#### General Molecular Characterization of DOM Leachates

The DOM leachates contain 2287–2784 unique molecular formulas with CHO compounds being the predominant species consisting of 82–86% of the total assigned molecular formulas (Table S5). The remaining 20% of formulas consist of carbon—hydrogen—oxygen—nitrogen (CHON), carbon—hydrogen—oxygen—sulfur (CHOS), and carbon—hydrogen—oxygen—nitrogen—sulfur (CHONS) assignments. The burned pine wood PyDOM leachate molecules contain a higher density of C=C double bonds and C=C unsaturation than the unburned pine wood DOM leachate molecules, as the charred pine wood PyDOM leachate contains lower molecular weight (MW) and oxygen-to-carbon (O/C) and hydrogen-to-carbon (H/C) atom ratios and higher nitrogen-to-carbon (N/C), double bond equivalent (DBE), aromaticity index (AI_mod_), and nominal oxidation state of carbon (NOSC) in comparison to the uncharred pine wood DOM leachate molecules (Table S5). The burned pine bark PyDOM is assumed to follow this relationship of containing molecules with a higher density of C=C double bonds and C=C unsaturation compared to its original unburned biomass, which would be consistent with other studies comparing unburned and burned biomass via (−)­ESI-FT-ICR-MS.^31,32^ The dominant compound class found among the unburned DOM leachate is lignin-like molecules followed by those that are tannin-like (Table S5). The charred pine bark PyDOM is unique as it contains a greater abundance of condensed aromatic carbon-like molecules compared to the uncharred and charred pine wood samples (15% versus ∼1%).

The pine wood DOM leachate, charred pine wood PyDOM leachate, and the charred pine bark PyDOM leachate contain clusters of CHOS molecular formulas at H/C ∼1.5 and O/C ∼0.3 (Figure S4). Unlike the uncharred and charred pine wood leachates, the charred bark PyDOM leachate is unique in containing a cluster of CHON molecular formulas at H/C ∼0.7 and O/C ∼0.6. Very few CHONS molecular formulas (<5% abundance) are present in the samples not exposed to glutathione and vary in their H/C and O/C ratios.

#### General Molecular Characterization of DOM Following GSH-Reaction

The GSH-reacted DOM leachates contain a similar range of unique molecular formulas (2277–2520) compared to the unreacted DOM leachates with CHO compounds also being the predominant species consisting of 82–90% of the total assigned molecular formulas (Table S5). Noticeably, the amount of CHONS formulas increased 10-fold in the charred pine wood and charred bark PyDOM leachates upon exposure to GSH increasing from 1% to 10% of the formula list. However, this trend is not observed in the uncharred pine wood GSH-reacted DOM leachate, which contains ∼5% of CHONS in both the unreacted and GSH-reacted eluates. Thus, the reaction with glutathione did not incorporate more sulfur-containing species. The greater increase in CHONS molecular formulas in the charred PyDOM leachates indicates that through pyrolysis the PyDOM molecules are likely functionalized in a manner that increases their reactivity toward GSH whereas uncharred wood is unreactive. The increase in CHONS molecular formulas may also be the result of the thiol-functional group in CHOS molecular formulas reacting with other molecular formulas (CHO, CHON, CHOS) or GSH in solution—that would account for the decrease in CHOS molecular formulas in the GSH-reacted leachates compared to the unreacted DOM leachates. Additionally, many of the nitrogen- and sulfur-containing molecular formulas in uncharred pine wood DOM leachate exist near the signal-to-noise threshold that may attribute to the variation in their abundances.

The CHONS molecular formulas are plotted on a Van Krevelen diagram (Figure 1) to visualize the 10-fold increase in CHONS molecular formulas in the GSH-reacted PyDOM samples (pink triangles) compared to the unreacted PyDOM samples (yellow squares). As depicted in Figure 1, the unreacted CHONS molecular formulas (yellow squares) in the unreacted DOM leachate generally plot in a scattered pattern on a Van Krevelen diagram. Though, there is an apparent clustering of the CHONS in the unreacted uncharred pine wood DOM leachate and charred pine wood PyDOM leachate at 0.1 < O/C < 0.5, 1.0 < H/C < 2.0, that may be residual proteinaceous material found in the pine wood from the synthesis of lignin.

**Figure 1:**
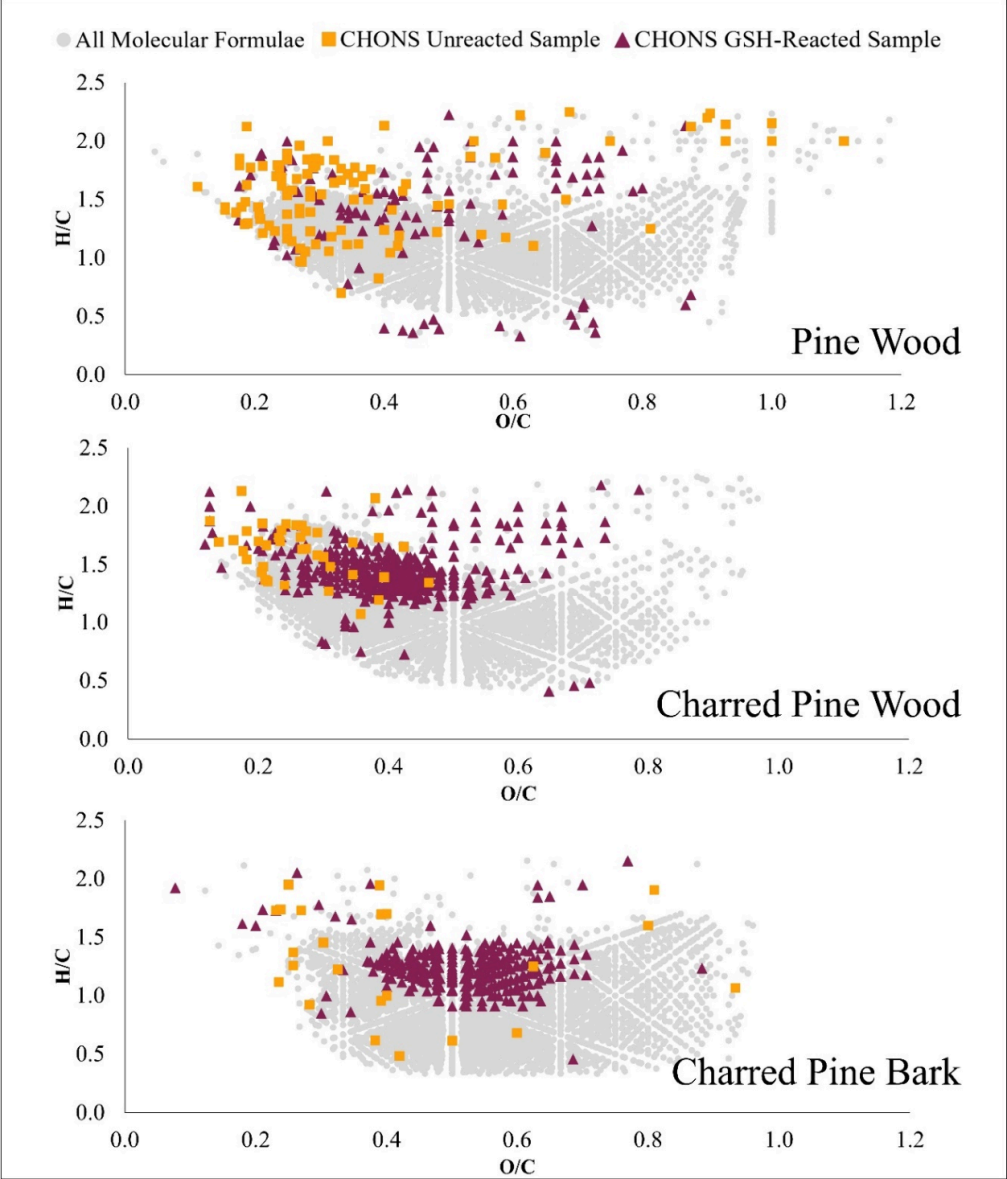
Van Krevelen diagram highlighting CHONS molecular formulas in the unreacted DOM leachates (yellow squares) and the GSH-reacted DOM leachates (pink triangles) and molecular formulas (gray circles) in both unreacted and GSH-reacted DOM leachates.

The GSH-reacted (pink triangles) CHONS molecular formulas from the uncharred pine wood DOM leachate forms a scattered set of formulas (0.2 < O/C < 0.4, 1.0 < H/C < 2.0). However, the CHONS molecular formulas from the GSH-reacted charred pine wood and bark PyDOM leachates plot as more focused and more populated clusters with similar H/C and O/C values, suggesting a distinct commonality of GSH structure incorporation due to increased chemical reactivity following pyrolysis.

#### KMD Analysis of Environmental Sample Set Leachates

KMD analysis is performed to elucidate plausible GSH interactions with the DOM leachate molecules (Supporting Information Section 4 for an analysis example). A nucleophilic attack by GSH on an electrophilic carbon is the most common reaction of GSH on saturated carbon atoms (e.g., alkyl halides, lactones, and epoxides), unsaturated carbon atoms (e.g., α,β unsaturated compounds, quinones, quinonimines, and esters), and aromatic carbon atoms (e.g., aryl halides and aryl nitro compounds). Two reaction types can be observed: addition-type KMD series with Kendrick mass ratio of 0.999727 and condensation/elimination-type reactions characterized by addition of GSH followed by loss of water with a Kendrick mass ratio of 0.999746. The KMD series is observed in Figure 2 by plotting the molecular formula pair’s Kendrick Nominal Mass (KNM) versus the Kendrick Mass Defect. Panel A indicates KMD pairs identified using addition-type Kendrick mass ratios, and panel B indicates KMD pairs identified using condensation/elimination-type Kendrick mass ratios. The precursor purple circles indicate molecular formulas proposed to form a covalent bond with GSH, while CHONS product pink triangles indicate the species produced following GSH-reacted DOM leachate molecules.

**Figure 2:**
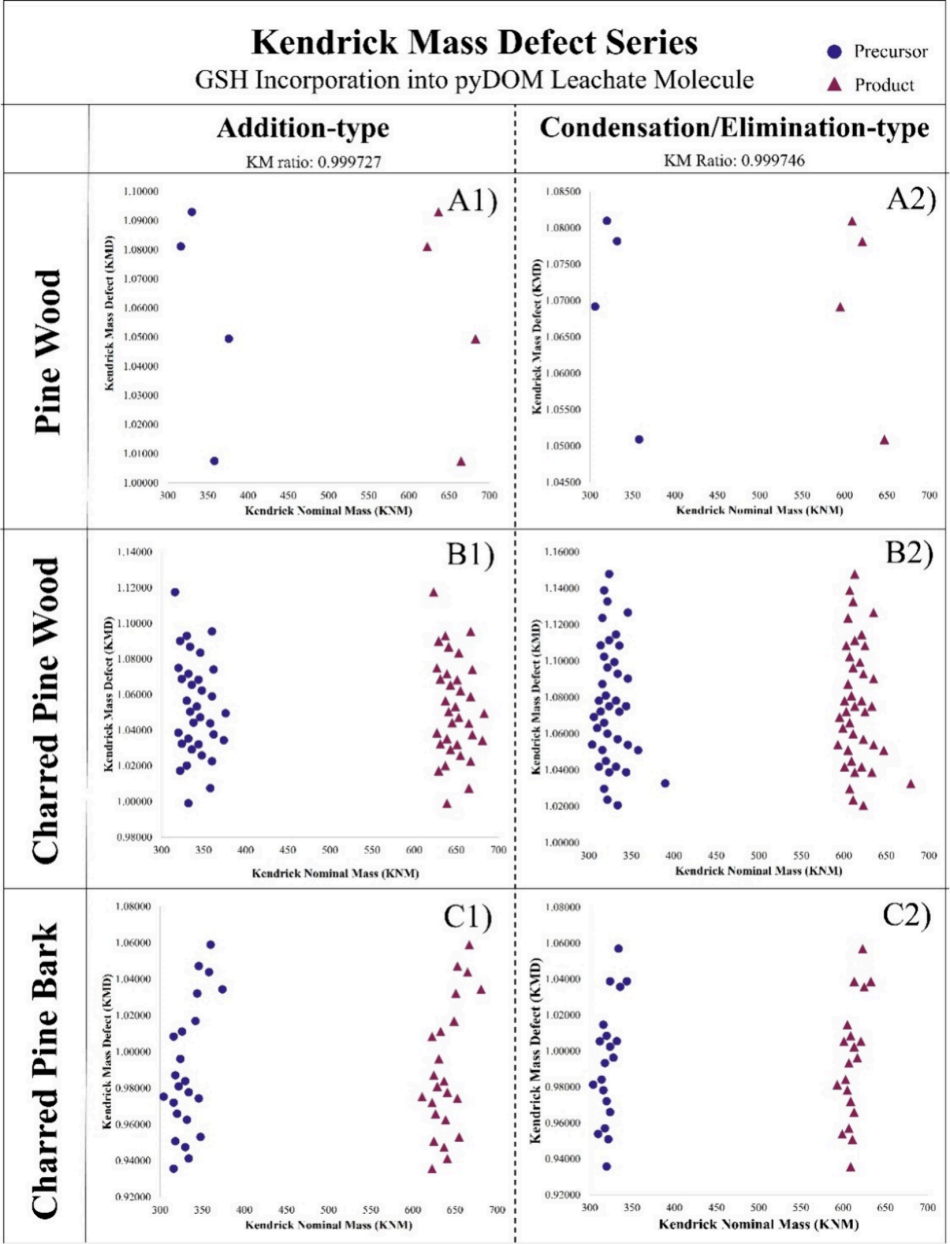
KMD series indicating molecular formulas in unreacted samples (precursor, purple circles) that reacted with GSH (CHONS products, pink triangles) for Pine Wood DOM leachate (A1–2), Charred Pine Wood PyDOM leachate (B1–2), and Charred Pine Bark PyDOM leachate (C1–2).

The GSH-reacted uncharred pine wood DOM leachate contains only 4 formula pairs associated in an addition-type KMD series and 4 formula pairs associated in an elimination/condensation-type KMD series, accounting for approximately eight percent of the CHONS formulas in the GSH-reacted DOM leachate (Figure 2, A1 and A2). On the other hand, the charred pine wood and charred bark PyDOM leachates contain more molecular formulas associated with a KMD series. The GSH-reacted charred pine wood PyDOM leachate contains 34 formula pairs associated in an addition-type KMD series and 40 pairs in an elimination/condensation-type KMD series, accounting for approximately 24% of the CHONS formulas in the sample (Figure 2, B1 and B2). The GSH-reacted charred pine bark PyDOM leachate is similar in this regard as it contains 23 formula pairs associated in an addition-type KMD series and 20 pairs in an elimination/condensation-type KMD series, accounting for approximately 17% of the CHONS formulas in the PyDOM leachate (Figure 2, C1 and C2).

The precursors are comprised exclusively of CHO molecules that ranged in molecular weight from 304–390 Da and oxygen content (3–9 oxygen present in molecular formulas). Most of the CHO precursor molecules contain AImod ≤ 0.5 and H/C < 1.5, indicating that the CHO precursors are likely highly unsaturated and phenolic compounds.^33^ There is overlap in the CHO precursor molecules found in the three GSH-reacted DOM samples. Fifteen of the KMD precursor molecules are found in the KMD series of at least two of the samples. These shared precursor molecules are found to have 6 in common between the uncharred pine wood DOM and charred pine wood PyDOM, 8 in common between the charred pine wood PyDOM and the charred pine bark PyDOM, and 1 common precursor CHO molecule between all three samples. These shared CHO precursors likely contain shared chemical structural motifs allowing for their ability to interact with GSH. However, it cannot be assumed that all molecular formulas in the DOM leachates share the same structural motifs. Twenty-two of the CHO precursor molecular formulas in the charred pine bark PyDOM are also identified in the uncharred pine wood DOM formula list; however, they do not form a new CHONS product. This highlights that though an equivalent molecular formula is found, the structure of that formula and its propensity to chemically react with GSH clearly varies; the charred PyDOM has a molecular composition that can chemically react, whereas a different isomer is likely present in the uncharred pine wood DOM, hence the lack of CHONS molecular formulas and KMD series pairs observed.

Overall, less than 25% of CHONS molecular formulas contribute to a KMD series. This may be due to the inability to confirm the associated precursor molecules (e.g., the precursor molecule is potentially outside of the detection window of 200–800 amu of the FT-ICR-MS). It is also plausible that CHON and CHOS molecules in situ formed new CHONS molecules. This is supported by the presence of two sulfur atoms in the CHONS molecular formulas in the charred pine bark PyDOM (17% of CHONS molecular formulas), charred pine wood PyDOM (20%), and uncharred pine wood DOM (45%), which may be the product of the formation of a disulfide bond. We suggest two plausible mechanisms for GSH to bond with molecules in the DOM leachates (SI Section 5) and recognize that other mechanisms of GSH incorporation (e.g., thiol–ene, thiol reactions with carbonyls to form hemithioacetals, thiol-alcohol interactions to produce sulfides)^34^ may be occurring in addition to other side reactions (e.g., cyclization).

The distribution and molecular characteristics of the GSH-incorporated formulae identified in the KMD series are visualized by plotting the molecular formula pairs on a van Krevelen diagram (Figure 3). The clustering pattern of the CHONS product molecular formulas seen in the diagram further supports that the precursor molecular formulas are covalently bonding to produce CHONS product molecular formula because a cluster of molecular formulas in a Van Krevelen diagram is indicative of compounds of similar structure and nature. In this case, the CHONS product cluster resides in close proximity to where GSH would plot (O/C of 0.6 and H/C of 1.7) and contains the appropriate number of sulfur and nitrogen atoms associated with the tripeptide. This clustering pattern also plots where most of the KMD series CHONS molecular formulas from the GSH-reacted charred PyDOM leachates plot. Furthermore, the CHONS GSH-reacted molecular formulas increase in their H/C and O/C ratio in comparison to their associated precursor molecules, that is observed on the van Krevelen as a diagonal shift toward the top right corner of the diagram.

**Figure 3:**
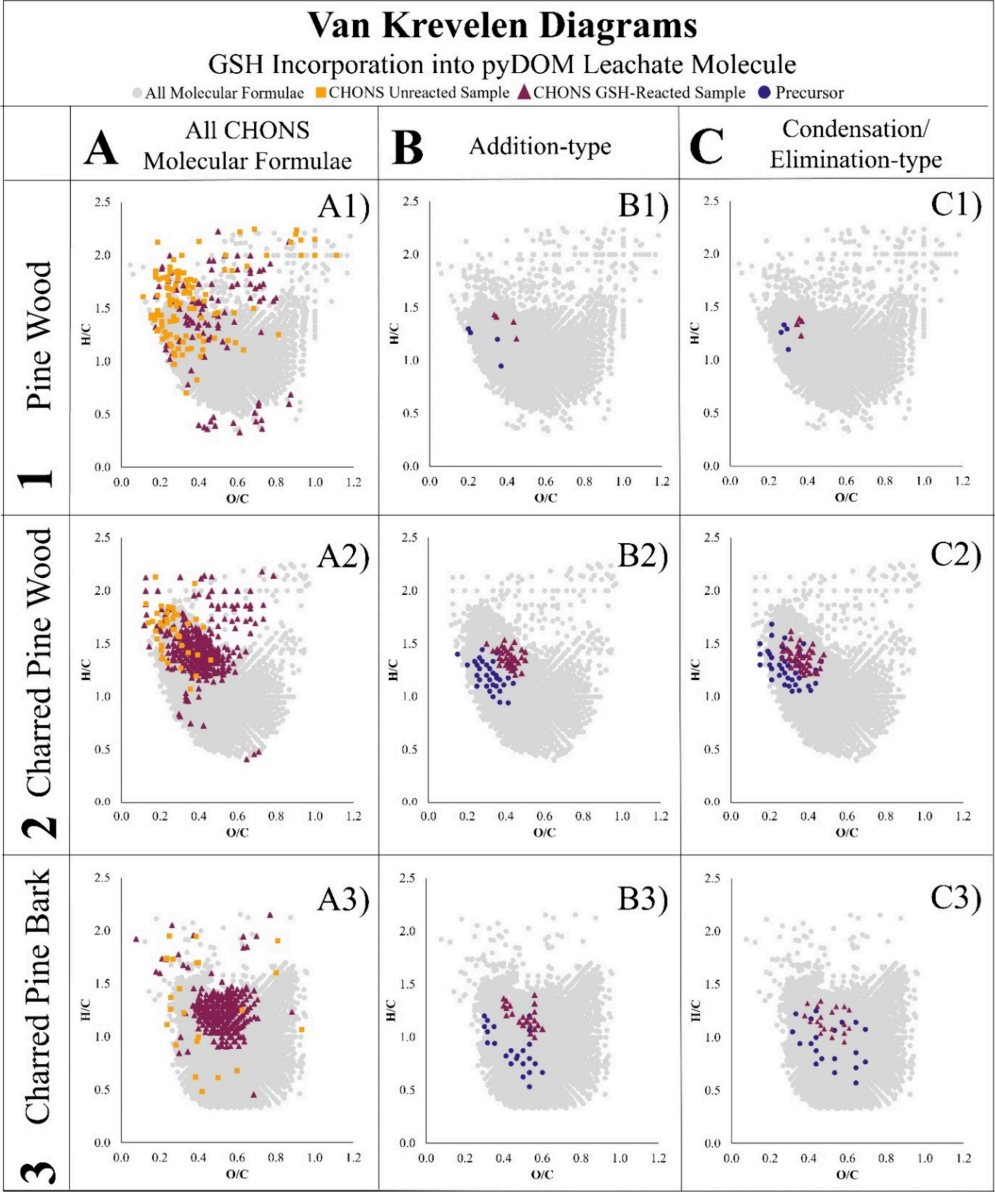
Van Krevelen diagrams of all CHONS molecular formulas (A), addition-type KMD series pathway (B), and condensation/elimination-type KMD series pathway (C) for environmentally weathered pine wood DOM leachate (1), charred pine wood PyDOM leachate (2), and charred pine bark PyDOM leachate (3).

#### Influence of Pyrolysis on Biomass Chemical Reactivity

Exposure to wildfire activity changes the chemical composition of the original biomass varying from slightly charred material to highly condensed graphitic sheets—often referred to as the combustion continuum.^35^ The functionalization of biomass during pyrolysis generates chemically reactive PyDOM molecules capable of forming covalent bonds with GSH, which otherwise would not have occurred. The functionalization of the charred material is apparent in the solid-state ^13^C NMR of the biomass (Figure S1) and the increase in chemical reactivity after wildfire exposure is supported with a greater increase in CHONS molecular formulas in the charred samples in comparison to its uncharred state via (−)­ESI-FT-ICR-MS analyses.^36^ Although we are limited in elucidating the exact structures of the identified precursor molecules through the analytical techniques employed in this study, the size and polarity (i.e., oxygen content in molecular formulas) suggests that they would be able to pass through the cellular membrane and subsequently interact with GSH intracellularly.

## Conclusion

(−)­ESI-FT-IRC-MS analysis indicates a 10-fold increase in nitrogen- and sulfur-containing molecular formulas in the GSH-reacted charred biomass PyDOM leachates compared to the unreacted charred biomass PyDOM leachates, which was not observed to nearly the same extent in the uncharred biomass GSH-reacted DOM leachate. This highlights the difference in chemical reactivity of wildfire-derived dissolved organic molecules versus uncharred dissolved organic molecules when introduced to GSH, making the benchtop GSH-reaction of PyDOM a promising probe for future toxicological studies. KMD analysis of the (−)­ESI-FT-ICR-MS formula lists attributed approximately 25% of the new nitrogen- and sulfur-containing molecules present in the GSH-reacted leachates as either an addition-type or condensation/elimination-type reaction occurring between the biomass leachate and GSH.

Future work is needed to investigate the toxicity of oxygen-containing PyDOM molecules on aquatic fauna, which may account for a missing component preventing the correlation of quantified PAHs to cellular death. Particularly, highly unsaturated and phenolic compounds in the 300–400 Da range with a large oxygen content (up to 9) should be considered, as indicated through this study’s KMD analysis. This is consistent with FT-ICR-MS and toxicology studies of biochar and hydrochar, that also indicate that pursing the impact of multiple oxygen-containing molecular formulas are of toxicological interest.^11–13^ This may be accomplished through an in vitro toxicology study correlating the depletion of GSH with molecular formulas identified from the benchtop GSH-reaction of PyDOM using the GSH KMD analysis. Employing the GSH KMD analysis may pose as a useful tool for preliminary screening of the potential uptake and exposure of dissolved PyC to higher organisms, that is important in evaluating overall ecosystem health due to the global presence, abundance, longevity, and propensity of wildfire occurrence—and PyC production—in the coming decades.

## Supplementary Material





## Notes

Additional experimental details, methods, and results (PDF)
Tables showing additional experimental details and results (XLSX)
