# Supplementary material for: Evaluating the Chemical Reactivity of Wildfire-Derived Dissolved Organic Molecules: Glutathione Binding through Kendrick Mass Defect Analysis
Source: J Am Soc Mass Spectrom. 2025 May 13;36(6):1377–85. doi: 10.1021/jasms.5c00077 (PMC12142673; doi:10.1021/jasms.5c00077)
Supplement: Supplementary file 1 [file js5c00077_si_001.pdf]

SUPPORTING INFORMATION – Evaluating the Chemical Reactivity of Wildfire-Derived Dissolved Organic Molecules: Glutathione Binding through Kendrick Mass Defect Analysis

Hannah M. Hamontree* and Patrick G. Hatcher**

Department of Chemistry & Biochemistry, Old Dominion University, 4501 Elkhorn Ave, Norfolk, Virginia 23529, United States of America;

*Phone: +1 813 454 7917; Email: hannah.hamontree96@gmail.com

**Phone: +1 757 683 6537. Email: phater@odu.edu

Section 1. Bulk characterization of Environmental Sample Set and DOM leachates…………2-3

Section 2. Spectroscopic structural analyses ……………………………………………………3-6

Section 2.1 Solid-state 13C nuclear magnetic resonance (NMR) ……………...………3-4

Section 2.2 One-dimensional (1D) 1H-NMR ………………………………………...5-6

Section 3. Molecular fingerprinting using Fourier transform - ion cyclotron resonance - mass spectrometry (FT-ICR-MS)…… ………………………….......................................……....7-10

Section 3.1 Instrumental Analysis……………………………………………….....….7

Section 3.2 Results………………………..……………………………………....8-10

Section 4. Kendrick Mass Defect Analysis & Proposed pathways for GSH covalent bonding to PyDOM leachates……………………………………………………….……….....11-12

Section 5. References…………………………...…………………………...……12-13

All supporting information tables are in the associated Microsoft Excel Worksheet (XLSX).

Section 1. Bulk characterization of Environmental Sample Set and DOM leachates

Elemental Analysis and Dissolved Organic Carbon

Homogenized samples were weighed (0.100 – 0.500 mg) into tin capsules (5 x 9 mm, CE Elantech) and run on a Thermo Finnigan Flash EA 1112 Series. The signal was externally calibrated against a nicotinamide certified standard (Thermo Scientific) to calculate carbon, nitrogen, and hydrogen contents in weight percent. Leachates and blank were acidified to pH ~2 with HCl. Dissolved organic carbon (DOC) concentrations were obtained using a Shimadzu TOC-V analyzer and quantified with a potassium hydrogen phthalate standard curve.

The environmentally weathered samples range in carbon content based on their exposure to wildfire-derived pyrolysis conditions (Table S1). Uncharred pine wood is comprised of less carbon overall with a C% of ~53% and an H/C of 1.30. Upon exposure to wildfire activity, the charred pine wood and bark samples contain greater C% because of the reactions producing condensed aromatic carbon structures occurring during the pyrolysis process. This resulted in an H/C of ~0.50 for both charred samples. A noticeable difference between the uncharred and charred samples is also observed following aqueous leaching. The uncharred pine wood leachate contains

approximately 20-fold more carbon compared to the charred pine wood and bark leachate carbon (1.56 and 2.28 ppm, respectively). The greater carbon content in the uncharred leachate is likely due to a predominance of water-soluble organic molecules found in the pine wood, which can readily dissolve. Conversely, the charred samples leach less carbon from their biomass as the molecules produced during the pyrolysis of the original biomass are likely less water-soluble (lower oxygen content, lower polarity aromatic structures).

Section 2. Spectroscopic structural analyses

Section 2.1 Solid-state ^{13}C Nuclear Magnetic Resonance (NMR)

Homogenized samples (uncharred pine wood, charred pine wood, charred pine bark) were analyzed using a multiple cross polarization (multiCP) magic angle spinning pulse sequence in a 4 mm rotor covered with a Kel-F cap. Experiments were conducted with ^{1}H resonating at 400 MHz and ^{13}C resonating at 100MHz and spun at the magic angle (54.7°C) at a frequency of 14 MHz on a Bruker Avance II spectrometer. A 1.0 s recycled delay was applied, and continuous pulses were optimized at 0.5 s for 1,600 scans. All spectra were calibrated to a glycine external standard (176.18 ppm). Relative contributions of major carbon moieties were obtained by normalizing spectral signal in-tensity by that of the entire spectral region (0.0 – 215.0 ppm). Spectra integrals include methylenic C (0.0 – 45.0 ppm), αC in peptides (45.0 – 60.0 ppm), alkyl-O carbon (60.0 – 95.0 ppm), anomeric C (95.0 – 110.0 ppm), aromatic C (110.0 – 145.0 ppm), aromatic-O carbon (145.0 – 165 ppm), and amide/carboxyl C (165.0 – 215.0 ppm).

The degree of chemical transformation of the PyC biomass from the source material is apparent through solid-state ^{13}C NMR (Figure S1) which indicates aromatization (particularly in the

condensed region of the spectra from 110 – 130 ppm) and aromatic oxygen-containing functional groups (155 pm) upon fire exposure.

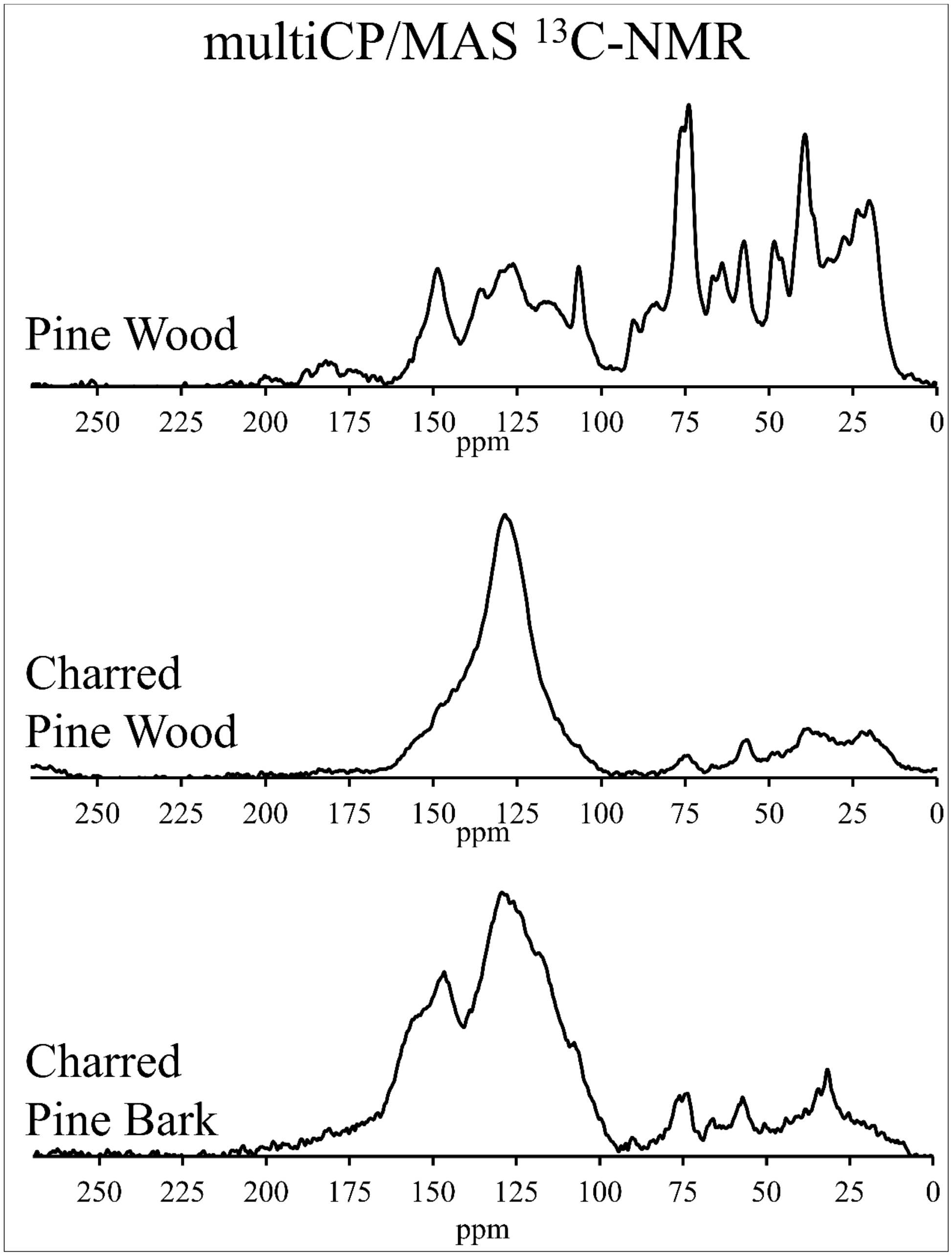

Figure S1. Solid-state ^{13}C NMR of environmentally exposed biomass samples indicating the structural changes (increased aromatic content 110- 155 ppm) which occur upon exposure to pyrolysis conditions.

Section 2.2 One-dimensional liquid ^{1}H Nuclear Magnetic Resonance (NMR)

Pine wood DOM leachate (Figure S2) is dominated by alkyl-C (0.60 – 1.80 ppm) and oxygenated-C (1.85 – 4.40 ppm) motifs which include methyl-h (H_3C-), methylene H (H_2C), H in alkyl groups that have heteroatoms bound to beta C (HC-C-CX, X= O,N,S), H bound to C that have an alpha C doubly bonded to C,O,N,S, (HC-C=y, carbonyl and carboxyl), H bound to C bound to N,S (HC-Z), carbohydrate and alcohol groups (HC-OR). The pine wood has some aryl-C (6.50 – 8.30 ppm) indicative of H attached to aromatic C (Ar-H). The charred sample DOM leachates have most of their signal in the HC-O-R region (3.20 – 4.40 ppm) indicating the presence of carbohydrate and/or alcohol groups and have sharp signals in the CH_2 (1.00-1.40 ppm), HC-C-C-C-X (1.40 – 1.85 ppm), HC-C=Y (1.85-3.2 ppm). Both samples contain aryl-C indicative of H attached to aromatic C (6.50 – 8.30 ppm).

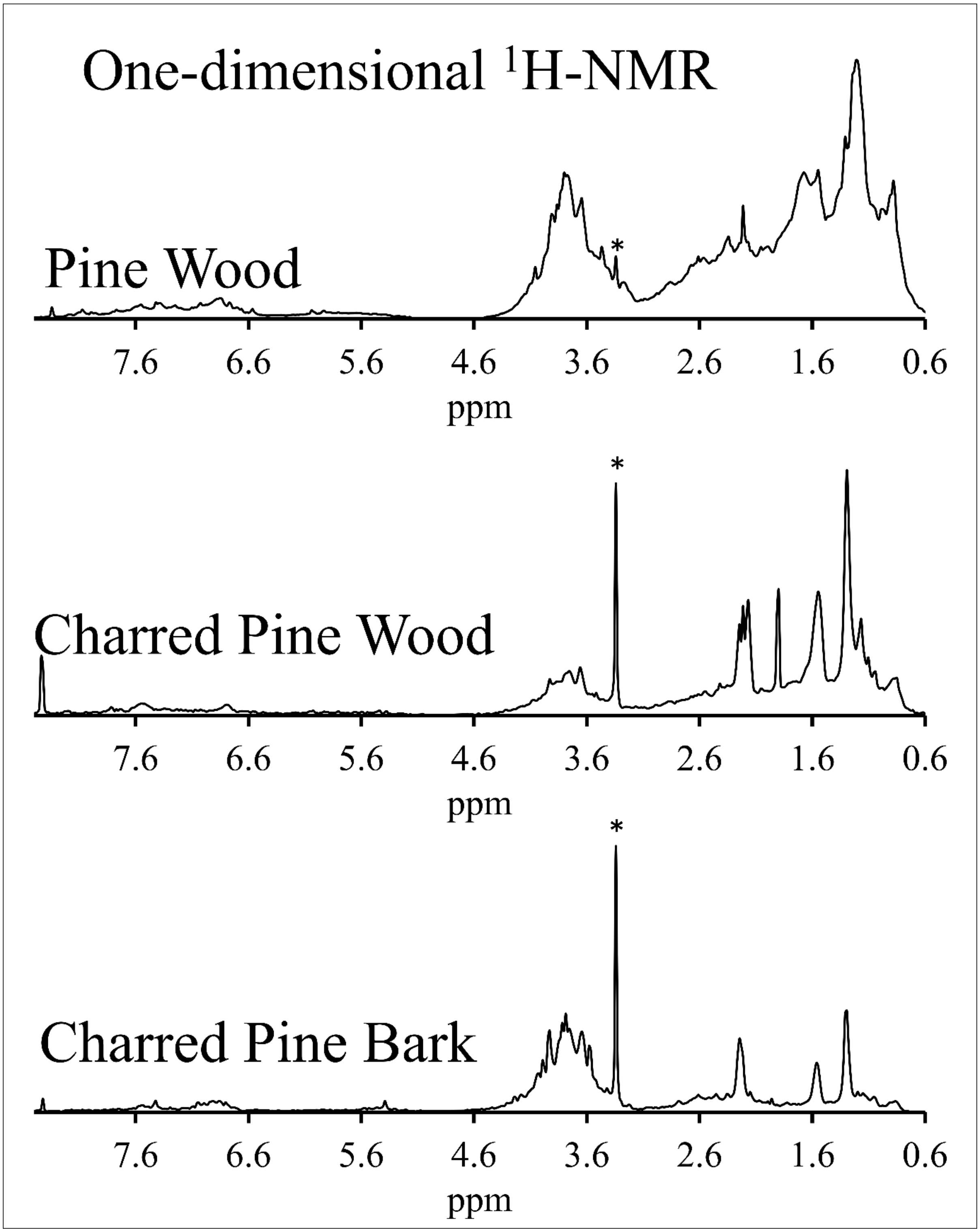

Figure S2. ^{1}H NMR of environmentally exposed biomass samples indicating the structural motifs of the respective DOM leachates. Methanol is denoted in the spectrum *

Section 3. Molecular fingerprinting using Fourier transform – ion cyclotron resonance – mass spectrometry (FT-ICR-MS)

Section 3.1 Instrumental Analysis

Ultrahigh resolution mass spectrometry allows one to identify exact masses from which unique elemental formulas can be extracted. Organic molecules typically found in PyDOM leachates are generally uncoupled from anything resembling GSH bonding (i.e., containing multiple oxygen, nitrogen, and sulfur heteroatoms in a single molecular formula). Molecular formulas with an increase in nitrogen and sulfur heteroatom content are products of the organic molecules in the DOM leachate forming new chemical bonds with GSH. As such, it is possible to elucidate new carbon-hydrogen-oxygen-nitrogen-sulfur-containing (CHONS) molecular formulas produced through the covalent bonding of GSH with soluble organic molecules in the DOM leachate. Under unbuffered reaction conditions at low pH, GSH creates covalent bonds that are more stable, less reversible, but form slower[1], thus affording the produced GSH-adducts greater likelihood of observation via FT-ICR-MS.

Section 3.2 Results

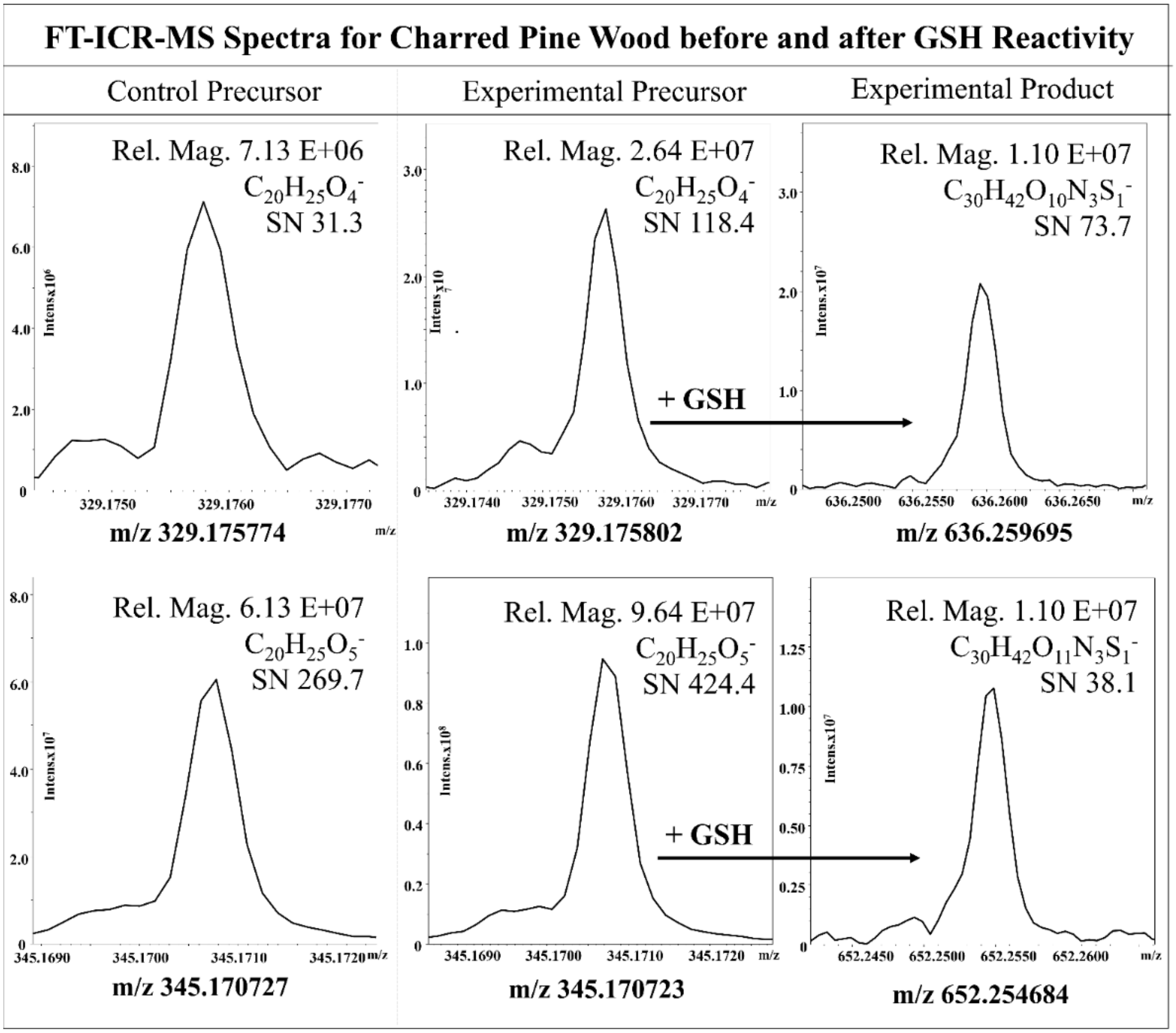

Figure S3. Example of (-)ESI-FT-ICR-MS spectra indicating peak shape, relative magnitude, assigned molecular formula, and signal to noise for unreacted (control) precursor sample and GSH-reacted (experimental) precursor and product sample.

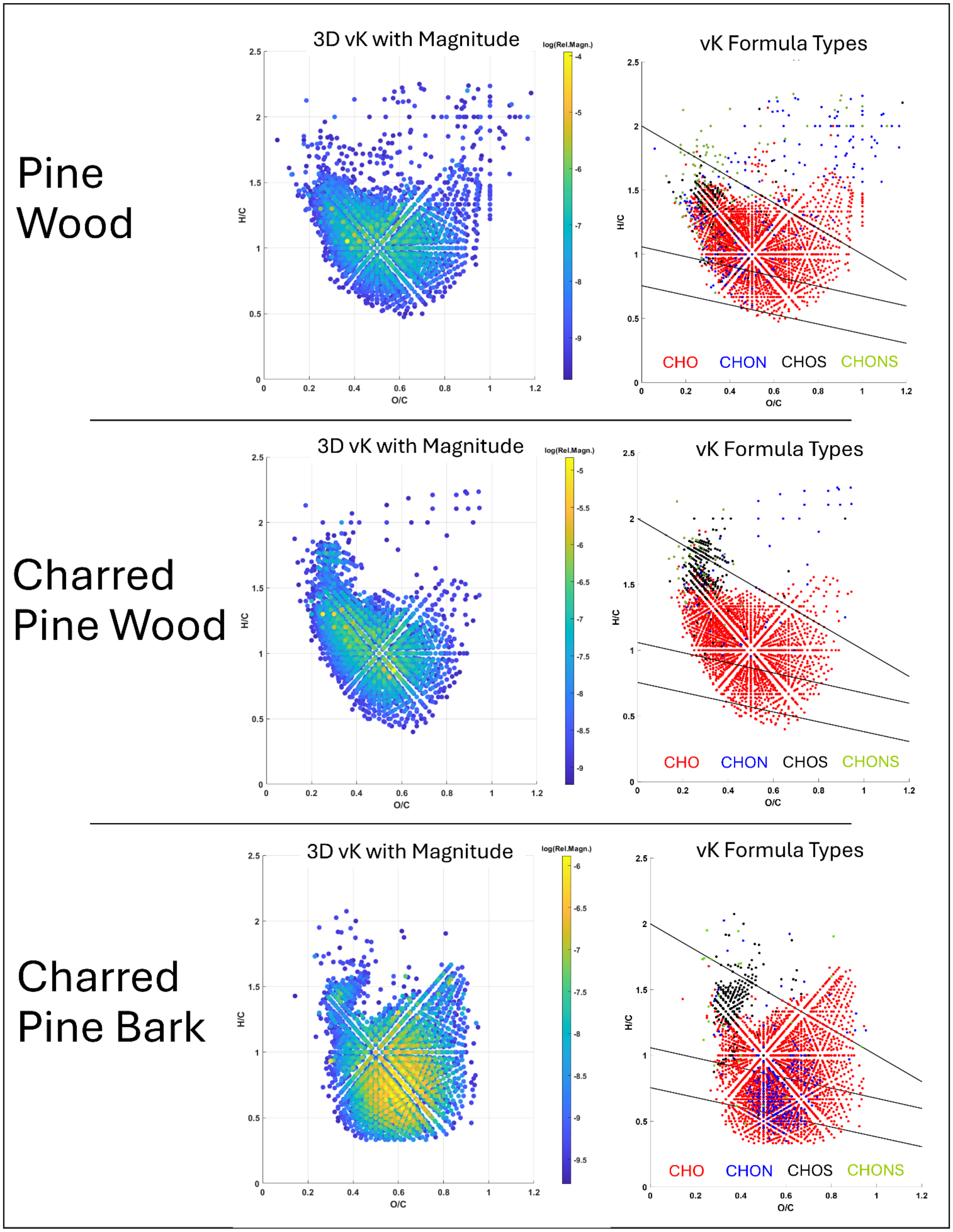

Figure S4. Van Krevelen diagram of biomass samples prior to GSH incorporation indicating magnitude and formula types of molecular formulas identified from the mass lists.

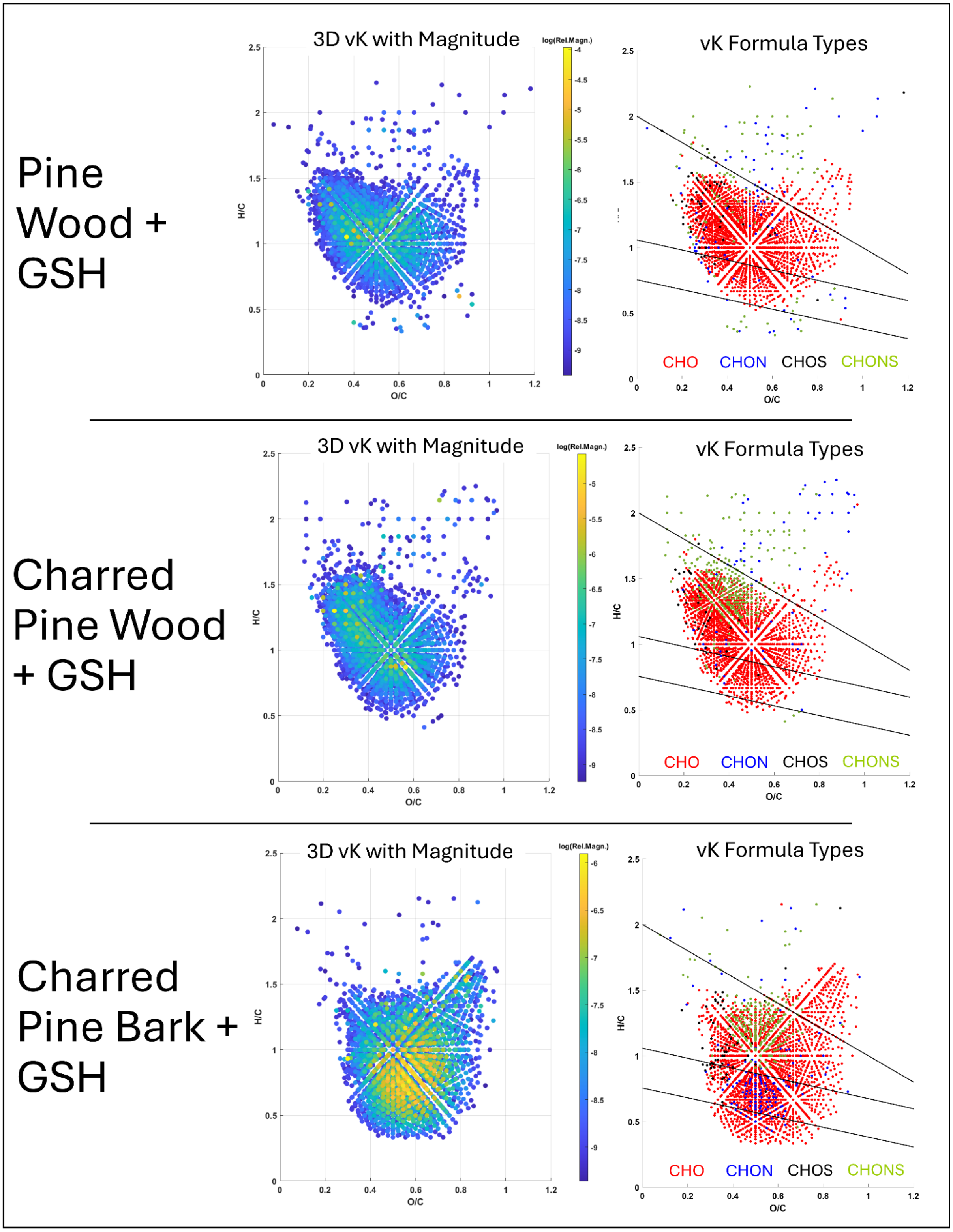

Figure S5. Van Krevelen diagram of biomass samples following GSH incorporation indicating magnitude and formula types of molecular formulas identified from the mass lists.

Section 4. Kendrick Mass Defect Analysis & Proposed pathways for GSH covalent bonding to PyDOM leachates

A CHO molecular formula was selected from the charred pine bark. The formula $C_{20}H_{26}O_5$ (m/z 346.178023) was identified in the FT-ICR-MS mass list of the charred wood and the theoretical structure proposed in Scheme S1 can undergo an addition-type reaction such as a thiol 1,4-Michael addition to form the observed CHONS product $C_{30}H_{43}O_{11}N_3S_1$ (m/z 653.261828), also found in the charred wood mass list. The theoretical structure was chosen only to demonstrate how two reaction pathways were plausible from the same chemical motif (quinone). The same precursor formula $C_{20}H_{26}O_5$ (m/z 346.178023) could undergo a condensation/elimination reaction such as a Schiff base reaction with GSH, with a subsequent loss of water, to form the observed CHONS product $C_{30}H_{41}O_{10}N_3S_1$ (m/z 635.251264) in the charred wood mass list. The implementation of a KMD analysis of the FT-ICR-MS molecular formulas verifies that the CHONS formulas are derived solely through the covalent bonding of GSH as it identifies repeating structural moieties between specific formula as either a thiol 1,4-Michael addition of glutathione (KMR 0.999727) or a Schiff base reaction (KMR 0.999746).

Although we are unable to report the definitive mechanistic pathway of GSH incorporation, we propose either a thiol 1,4-Michael addition or Schiff base as likely mechanisms for the GSH covalent bonding to PyDOM leachate molecules. These mechanistic pathways are plausible as oxidation-reduction type, nucleophilic displacement, and addition type reactions are known to occur with GSH activity in cells[2–4]. It is likely that a 1,4-Michael addition dominates as the thiol group is a soft nucleophile and would chemically react with the β-carbon of the electrophile since it is significantly softer than the carbonyl carbon[5]. Thiol addition to *o*-quinones selectively prefers the carbon directly adjacent to the o-quinone to form the adduct and is directed by the lower

carbonyl, as shown in Scheme S1 [6,7]. An amino 1,4-Michael adduction is also possible, however, the thiol adduction is the highly preferred reaction, as thiols are more nucleophilic and more polarizable.

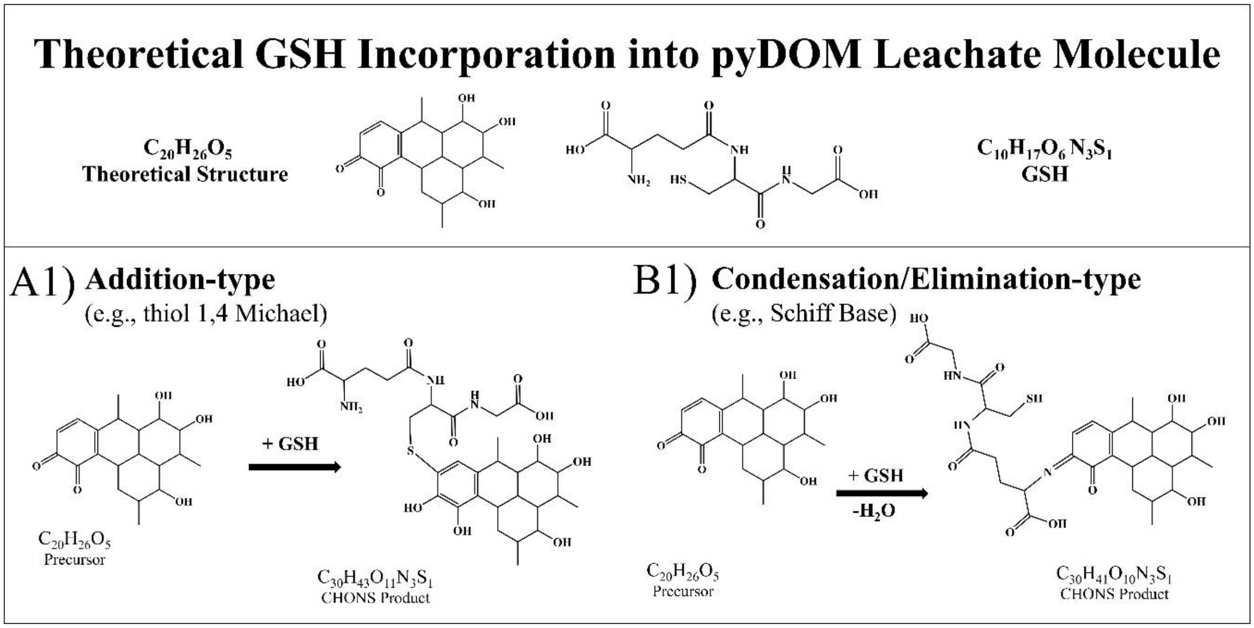

Scheme S1. Proposed pathways for GSH bonding to PyDOM leachate molecules via thiol 1,4-Michael addition (A1) or Schiff base (B1).

Section 5. References

1. Schmidt, T. J., Lyû, G., Pahl, H. L., Merfort, I.: Helenanolide Type Sesquiterpene Lactones. Part 5: Y The Role of Glutathione Addition Under Physiological Conditions. *Bioorg. Med. Chem*. **7**, 2849-2855 (1999)

2. Meister, A., Tate, S. S.: Glutathione and Related Y-Glut Amyl Compounds: Biosynthesis and Utilization. *Annu. Rev. Biochemistry* **45**, 559–604 (1976)

3. DeLeve, L. D., Kaplowitz, N.: Glutathione Metabolism and Its Role in Hepatotoxicity. *Pharmacol. Ther.* **52**, 287–305 (1991)

4. Gilbert, H. F.: Biological Disulfides: The Third Messenger? Modulation of phosphofructokinase activity. *J. Biol. Chem.* **257**, 12086-12091 (1982)

5. Böhme, A., Thaens, D., Paschke, A., Schüürmann, G.: Kinetic Glutathione Chemoassay to Quantify Thiol Reactivity of Organic Electrophiless - Application to α,β-Unsaturated Ketones, Acrylates, and Propiolates. *Chem. Res. Toxicol.* **22**, 742–750 (2009)

6. Alfieri, M. L., Cariola, A., Panzella, L., Napolitano, A., d'Ischia, M., Valgimigli, L., Crescenzi, O.: Disentangling the Puzzling Regiochemistry of Thiol Addition to O-Quinones. *J. Org. Chem.* **87**, 4580–4589 (2022)

7. Kishida, R., Ito, S., Sugumaran, M., Arevalo, R. L., Nakanishi, H., Kasai, H.: Density Functional Theory-Based Calculation Shed New Light on the Bizarre Addition of Cysteine Thiol to Dopaquinone. *Int. J. Mol. Sci.* **22**, 1373 (2021)
